# Microstructure of the Bonnethead Shark (*Sphyrna tiburo*) Olfactory Rosette

**DOI:** 10.1093/iob/obac027

**Published:** 2022-07-18

**Authors:** Lauren E Simonitis, Christopher D Marshall

**Affiliations:** Department of Marine Biology, Texas A&M University, Galveston Campus, Galveston, TX 77553, USA; Department of Marine Biology, Texas A&M University, Galveston Campus, Galveston, TX 77553, USA; Department of Ecology and Conservation Biology, Texas A&M University, College Station, TX 77843, USA

## Abstract

**Synopsis:**

The unusual shape of sphyrnid (hammerhead shark) heads has led to many functional hypotheses of potential sensory advantages and enhanced olfactory performance. Recent investigations into the flow of water within the sphyrnid olfactory chamber demonstrate that this complex structure exhibits a differential pressure system between the 2 nares that induces flow through the chamber. This leads to differential fluid velocities in different parts of the olfactory chamber. Particularly, lamellae at the medial end of the olfactory chamber experience a near-stagnant recirculation of water. The objectives of this study were to (1) describe the microstructure of the olfactory rosette of bonnethead sharks (*Sphyrna tiburo*) and (2) given the variability of water flow within the sphyrnid olfactory rosette, investigate differences of individual lamellae based on their positioning within the rosette. Specifically, we investigated degree of secondary folding, percent sensory area, and relative surface along the lateral-to-medial gradient. Both degree of secondary folding and percent sensory area may serve as proxies for olfactory sensitivity, providing connectivity between area devoted to sensitivity and water flow within the olfactory organ. We found that bonnethead sharks exhibited similar lamellar morphology to other shark species. We also described the projection of the olfactory nerve layer through an individual lamella. Additionally, we found that lamellae within the medial portion of the organ, which experience slower water velocities, had less secondary lamellar folds and less sensory area. These findings imply that these areas may be less sensitive. Future work should test for sensitivity differences within the rosette along the lateral-to-medial gradient.

**Spanish:**

La forma inusual de las cabezas de los esfírnidos (tiburones martillo) ha llevado a muchas hipótesis funcionales de posibles ventajas sensoriales y unas mejores capacidades olfativas. Las investigaciones recientes sobre el flujo de agua dentro del órgano olfativo de los esfírnidos, demuestran que esta estructura compleja exhibe un sistema de presión diferente entre las dos fosas nasales que induce el flujo en el órgano. Esto conduce a velocidades de fluido diferentes en distintas partes del órgano olfativo. En particular, las láminas en el extremo medial del órgano olfativo experimentan una recirculación de agua casi estancada. Los objetivos de este estudio fueron 1) describir la microestructura de la roseta olfativa de los tiburones cabeza de pala (Sphyrna tiburo) y 2) considerando la variabilidad del flujo de agua dentro de la roseta olfativa de los esfírnidos, investigar las diferencias de las laminillas individuales, basadas en su posición dentro de la roseta. Específicamente, hemos investigado el grado de plegamiento secundario, el porcentaje del área sensorial y el área relativa de superficie a lo largo del gradiente de lateral a medial. El grado de plegamiento secundario y el porcentaje del área sensorial pueden servir como indicadores de la sensibilidad olfativa, proporcionando conectividad entre el área dedicada a la sensibilidad y el flujo de agua dentro del órgano olfativo. Descubrimos que los tiburones cabeza de pala exhibían una morfología laminar similar a la de otras especies de tiburones. También hemos descrito la proyección del estrato del nervio olfativo dentro de una lámina individual. Además, encontramos que las laminillas dentro de la porción medial del órgano que experimentan velocidades de agua más lentas, tenían menos pliegues laminares secundarios y una menor área sensorial. Estos hallazgos implican que estas áreas pueden ser menos sensitivas. El trabajo futuro debería evaluar las diferencias de sensibilidad dentro de la roseta a lo largo del gradiente de lateral a medial.

**German:**

Die ungewöhnliche Kopfform der Sphyrniden (Hammerhaie) hat schon zu vielen funktionellen Hypothesen bezüglich möglicher sensorischer Vorteile und verbesserter olfaktorischer Leistung geführt. Kürzlich veröffentlichte Studien zur Wasserströmung innerhalb der olfaktorischen Kammern von Sphyrniden zeigen, dass diese komplexe Struktur unterschiedliche Drucksysteme zwischen den beiden Nasenlöchern erzeugt, welches eine Strömung durch die Nasenkammer erzeugt. Dies wiederum führt zu unterschiedlichen Flüssigkeitsströmungen in verschiedenen Abschnitten der olfaktorischen Kammer. Besonders bei den Lamellen am medialen Ende der olfaktorischen Kammer gibt es eine fast schon stillstehende Rezirkulation von Wasser. Die Ziele dieser Studie waren 1) das Beschreiben der Mikrostruktur der olfaktorischen Rosette des Schaufelnasen-Hammerhais (*Sphyrna tiburo*) und 2) wollten wir, aufgrund der Variabilität der Wasserströmung innerhalb der olfaktorischen Rosette der Sphyrniden, die Unterschiede von individuellen Lamellen basierend auf ihrer unterschiedlichen Position innerhalb der Rosette untersuchen. Wir untersuchten den Grad an sekundären Falten, den Prozentsatz an sensorischer Fläche und die relative Oberfläche entlang dem lateral-zu-medialem Gradienten. Sowohl der Grad an sekundären Falten wie auch der Prozentsatz an sensorischer Fläche mögen als Annäherung für die olfaktorische Sensibilität dienen, weil sie für eine Verbindung zwischen der Fläche, die dem Geruchssinn und der Strömung zwischen den olfaktorischen Organen sorgt. Wir fanden, dass die Schaufelnasen-Hammerhaie eine ähnliche lamellare Morphologie zeigen wie andere Hai-Arten. Wir beschreiben auch wie der Geruchsnerv durch eine individuelle Lamelle verläuft. Weiter fanden wir, dass die Lamellen innerhalb des mittleren Teils des Organs, welches geringe Strömungsgeschwindigkeiten erfährt, weniger sekundäre lamellare Falten enthält und weniger sensorische Fläche. Diese Entdeckungen implizieren, dass diese Bereiche weniger sensibel sind auf Gerüche. Zukünftige Arbeiten sollten die unterschiedlichen Sensibilitäten innerhalb der Rosette entlang des lateral-medialem Gradienten testen.

## Introduction

Sharks have often been regarded as “swimming noses” with superior smelling ability by the popular media. However, physiological testing of the shark olfactory systems has shown that sharks are not any better at smelling than their bony fish cousins—sharks and teleosts exhibit similar olfactory capabilities (Meredith and Kajiura 2010). While the olfactory sensitivities of sharks and teleosts are similar, the morphology of their olfactory structures differs in olfactory bulb shape and location as well as olfactory sensory receptor type (Reese and Brightman 1970; Theisen et al. 1986; Zeiske et al. 1986, 1987; Caprio 1988; Lisney and Collin 2006; Zielinski and Hara 2006; Schluessel et al. 2008; Camilieri-Asch et al. 2020b). Olfactory morphologies also differ among sharks, which include variations in olfactory bulb insertion and olfactory peduncle length, lamellar surface area, and structure of the nares and olfactory rosettes (Smeets 1998; Kajiura et al. 2005; Schluessel et al. 2008; Meredith and Kajiura 2010; Yopak et al. 2015). These differences are related to species-specific neuroecology rather than differences in sensitivity or phylogeny (Schluessel et al. 2008; Meredith and Kajiura 2010; Yopak et al. 2015).

**Fig. 1:**
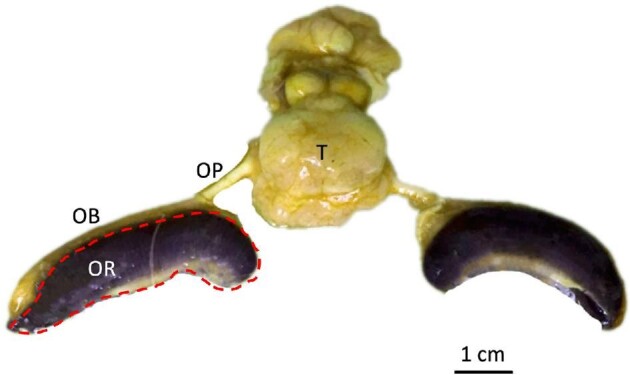
Gross anatomy of the bonnethead shark (*S. tiburo*) olfactory system. OB: olfactory bulb, OP: olfactory peduncle, OR: olfactory rosette (outlined in red), T: telencephalon.

**Fig. 2:**
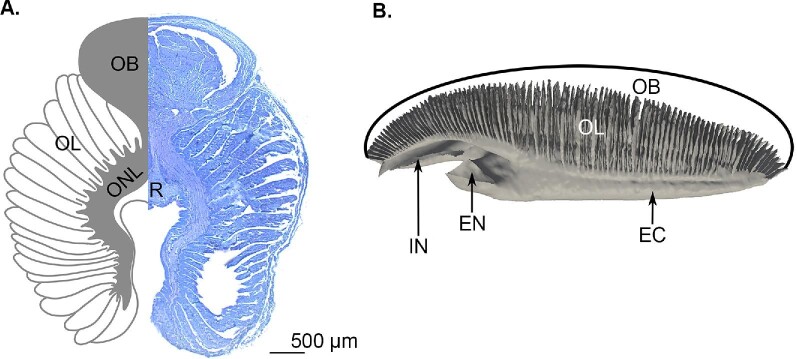
Morphology of two sphyrnid olfactory organs. (**A**) A cross section through the olfactory rosette of a bonnethead shark and (**B**) the 3D anatomy of the olfactory rosette from a smalleye hammerhead shark (Rygg et al. 2013). EC: excurrent canal, EN: excurrent naris, IC: incurrent canal, IN: incurrent naris, OB: olfactory bulb, OL: olfactory lamellae, ONL: olfactory nerve layer, R: raphe. **A** stained with toluidine blue.

Sharks have an incurrent and excurrent naris to allow for unidirectional water flow. As a shark swims, water passively travels through the incurrent naris and into the incurrent canal of the olfactory rosette (Figs. 1 and 2). This canal extends through and across the rosette and its many lamellae. Within the incurrent canal, water passes over the lamellae and flows in between their secondary folds, which are covered by olfactory epithelium (Zeiske et al. 1987). Lamellar secondary folds have been shown to increase surface area 70–495% (Ferrando et al. 2019). Chemicals dissolved in the water (solvents) bind to G-protein-coupled molecular receptors on olfactory receptors neurons (ORNs) on the surface of the olfactory epithelium (Smeets 1998; Eisthen 2004). Water then flows out through the excurrent canal, passing the posterior ends of the lamellae, and out the excurrent naris (Zeiske et al. 1987; Abel et al. 2010; Rygg et al. 2013).

The olfactory epithelium is divided into nonsensory and sensory components. While a patchy, irregular distribution of epithelium has been described for one shark species, Port Jackson sharks (*Heterodontus portusjacksoni*), other described species have nonsensory epithelium concentrated at the inner margins of lamellae and sensory epithelium extending almost to the outer margin (Schluessel et al. 2008; Camilieri-Asch et al. 2020a). The nonsensory epithelium is comprised of goblet cells and is covered by small microvilli. The sensory epithelium contains the receptor cells, ciliated supporting cells, basal cells, and goblet cells (Zeiske et al. 1987; Schluessel et al. 2008; Meredith et al. 2013). The ciliated supporting cells are thought to move mucus or water across the olfactory epithelium. It is more likely that cilia influence mucus movement more so than water since Zeiske et al. (1987) reported no net water movement in the olfactory organ in stationary lemon sharks (*Negaprion brevirostris*). Additionally, Cox (2013) provided further evidence for cilia acting as mucus transporters due to observed water currents in the nasal region, mucociliary interactions, and ciliary morphometrics, movement, and distribution.

Underneath the olfactory epithelium is an olfactory nerve layer consisting of the ORN axons originating in the sensory epithelium (Fig. 2A). The axons of ORNs diverge within the olfactory bulb and then synapse with mitral cells (second-order olfactory neurons) within the glomeruli. Glomeruli are spherical structures, functionally similar to the ganglia of the peripheral nervous system, and are distributed throughout the bulb (Butler and Hodos 2005; Meredith et al. 2013). The axons of these mitral cells form part of the olfactory peduncles or tracts that communicate sensory information from the olfactory bulb to the telencephalon (Fig. 1; Laberge and Hara 2001; Yopak et al. 2015). The olfactory peduncles are most commonly known as cranial nerve I, the olfactory nerve.

The unusual shape of sphyrnid heads has led to many hypotheses regarding their function and potential sensory advantages. Sphyrnids are interesting because of the range of cephalofoil expansion within the group, with bonnethead sharks (*Sphyrna tiburo)* having the most truncated cephalofoil and winghead sharks (*Eusphyra blochii*) sporting the most elongated heads. Electrosensory wise, the head shape of sphyrnids has been hypothesized to provide a greater lateral search area and allow for enhanced maneuverability (Kajiura and Holland 2002). Within these elongated heads, sphyrnids also have larger, longer olfactory organs compared to other elasmobranchs, leading to hypotheses that they possessed superior smelling abilities. However, physiological testing has revealed that olfactory sensitivity of tested sphyrnids is comparable to other elasmobranchs (Tricas et al. 2009; Meredith and Kajiura 2010). Despite a lack of physiological superiority compared to other elasmobranchs, the sphyrnid olfactory morphology has been linked to some olfactory advantages. The spacing of the nares on either side of the head allows for greater separation between olfactory organs. This may provide sphyrnids with an advantage for odor localization and gradient navigation compared to their relatives with closer spaced nares (Tester 1963; Hasler, 1957; Kajiura et al. 2005). Additionally, the presence of prenarial grooves in most sphyrnids allow them to sample a higher volume of water. (Tester 1963; Kajiura et al. 2005; Rygg et al. 2013).

Recent investigations of water flow through the olfactory structures (Abel et al. 2010; Rygg et al. 2013) demonstrate that this complex morphology exhibits a differential pressure gradient between the two nares. This induces flow through the olfactory chamber. Additionally, sphyrnid olfactory structures regulate flow internally via the gaps between the lamellae, which are hypothesized to function as a partial bypass for water flow (Rygg et al. 2013). This leads to differential fluid velocities in different regions of the olfactory chamber. In smalleye hammerheads (*Sphyrna tudes*), lamellae at the medial end of the olfactory chamber experience a near-stagnant recirculation of water (Rygg et al. 2013). What are the sensory implications for this region of stagnation? Does lamellar morphology vary based on what velocity of water flow they experience?

The objectives of this study were (1) to describe the microstructure of the olfactory rosette of another sphyrnid, bonnethead sharks (*S. tiburo*) and (2) given the variability of water velocity along the sphyrnid olfactory rosette, to investigate morphological differences between individual lamellae based on their positioning within the rosette. Specifically, we investigated degree of secondary folding, percent sensory area, and relative surface area. Both degree of secondary folding and percent sensory are two morphological metrics that may serve as proxies for sensitivity. Although Meredith and Kajiura (2010) showed that sharks with larger lamellar surface area do not experience greater olfactory acuity, these studies focused on overall lamellar area, not sensory area. Because the olfactory receptor neurons (ORNs) are housed in the sensory epithelium, an increased sensory area may correlate with more ORNs and thus higher sensitivity. Secondary folds within the sensory epithelium increase surface area, allowing more space for ORNs. Additionally, size and density of neurological structures are often used as proxies for sensitivity (George and Holliday 2013; Jones and Marshall 2019). Both of these metrics provide connections between sensitivity and water flow within the olfactory organ. We hypothesized that the sensory morphology (amount of sensory surface area and number of secondary lamellar folds) would vary along the lateral-to-medial gradient correlating to differences in water flow within the olfactory organ.

## Materials and methods

### Sample collection

Four bonnethead shark specimens (*S. tiburo*) were donated by local fisherman caught from the waters surrounding Galveston, TX, USA. Olfactory systems including brains, olfactory tracts, and rosettes were removed immediately upon receipt of specimens and fresh fixed in 10% buffered formaldehyde. These structures were kept intact and connected to preserve orientation.

### Scanning electron microscopy (SEM)

To describe the surface of each lamella, rosettes (N = 3) were prepared for scanning electron miscroscopy. Each rosette was cut longitudinally, separating the right and left lamellae. Then, if possible, every third lamella was excised. In some cases, the third lamella was damaged, so the next undamaged lamella was taken. Lamellae were dehydrated using an ethanol dehydration series (30, 50, 70, 70, 80, 80, 95, 95, 100%) for 10 min per bath. Lamellae were then further dehydrated with Hexamethyldisilazane (HMDS) for 30 s, then removed and left to air dry on a paper towel. Dried lamellae were mounted on carbon stubs (Ted Pella, Inc, Redding, CA, USA) and sputter coated with gold/palladium before they were imaged in a Hitachi TM3000 (vacuum high, 15k acceleration voltage; Hitachi, Tokyo, Japan) or JEOL JCB 2000 scanning electron microscope (Vacuum high, 10k acceleration volage; JEOL USA Inc, Peabody, MA, USA).

### Light micrography (LM)

**Fig. 3:**
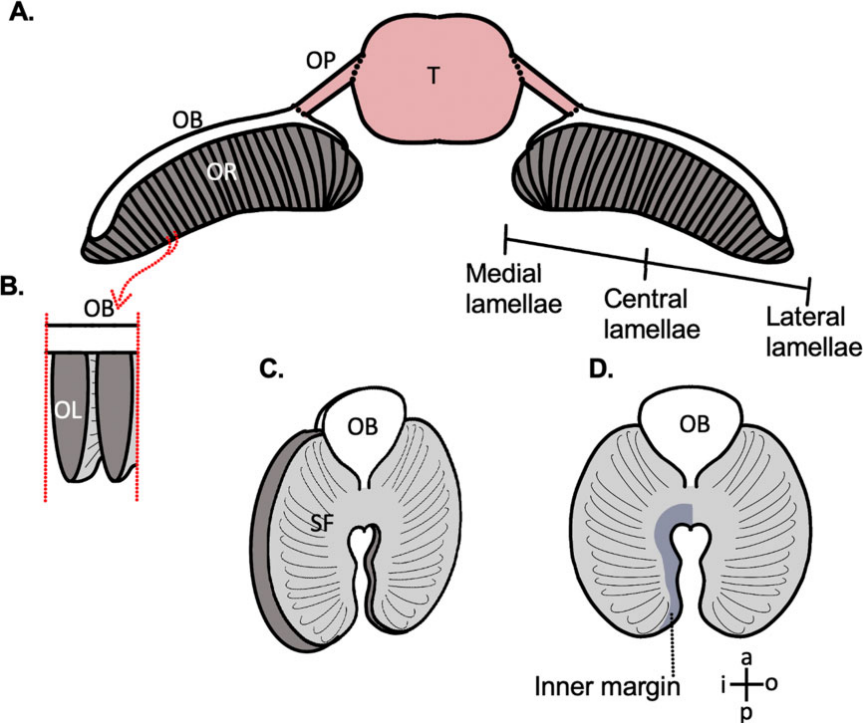
Anatomical atlas. (**A)** A schematic of the bonnethead shark (*S. tiburo*) olfactory system drawn from Fig. 1 showing lamellar positioning on the lateral-to-medial gradient. OB: olfactory bulb, OP: olfactory peduncle, OR: olfactory rosette, T: telencephalon. (**B**) Two OL isolated from the rosette attached to the olfactory bulb. (**C**) A schematic of a single lamella (angled) attached to the olfactory bulb depicting secondary folds (SF). (**D**) A single lamella (in cross section) attached to the olfactory bulb. Inner margin shaded. Lamellar directional terminology: anterior (a), posterior (p), inner (i), and outer (o).

One rosette was prepared histologically for LM to describe the internal microstructure of the lamellae. The rosette was dissected into 10 transverse sections, each containing both sides of the lamellae and the olfactory bulb (Figs. 2B and 3). Rosette sections were processed for paraffin histology using a Leica tissue processor (Leica Biosystems Inc., Buffalo Grove, IL, USA) under vacuum. Samples were processed through a dehydration series of alcohol (70, 80, 95, 95, 95, 100, 100, 100% for 1 h each), followed by infiltration of xylene (two baths for 1 h each) and paraffin (two baths for 1 h each). Samples were then embedded for cross-sectioning in a paraffin block and sectioned at 7 μm on a rotary microtome, keeping every fourth section. Sections were mounted onto 1% gel subbed slides and stained with a general stain, Toluidine Blue (Sigma-Aldrich, St. Louis, MO, USA). Stock 1% Toluidine Blue solution was made in 70% EtOH. Slides were deparaffinized in Xylene, rehydrated (100, 100, 95, 85, 70%), placed in a toluidine blue working solution (30 mL of stock Toluidine Blue solution and 270 mL of 1% NaCl solution) for 3 min, dehydrated (70, 85, 95, 100, 100%, Xylene), and cover slipped. Digital micrographs were collected using a Nikon E-400 (Nikon Instruments Inc., Melville, NY, USA) Eclipse light microscope fitted with a Spot Insight (Diagnostic Images, Sterling Heights, MI, USA) digital microscopy camera.

### Image analysis

**Fig. 4:**
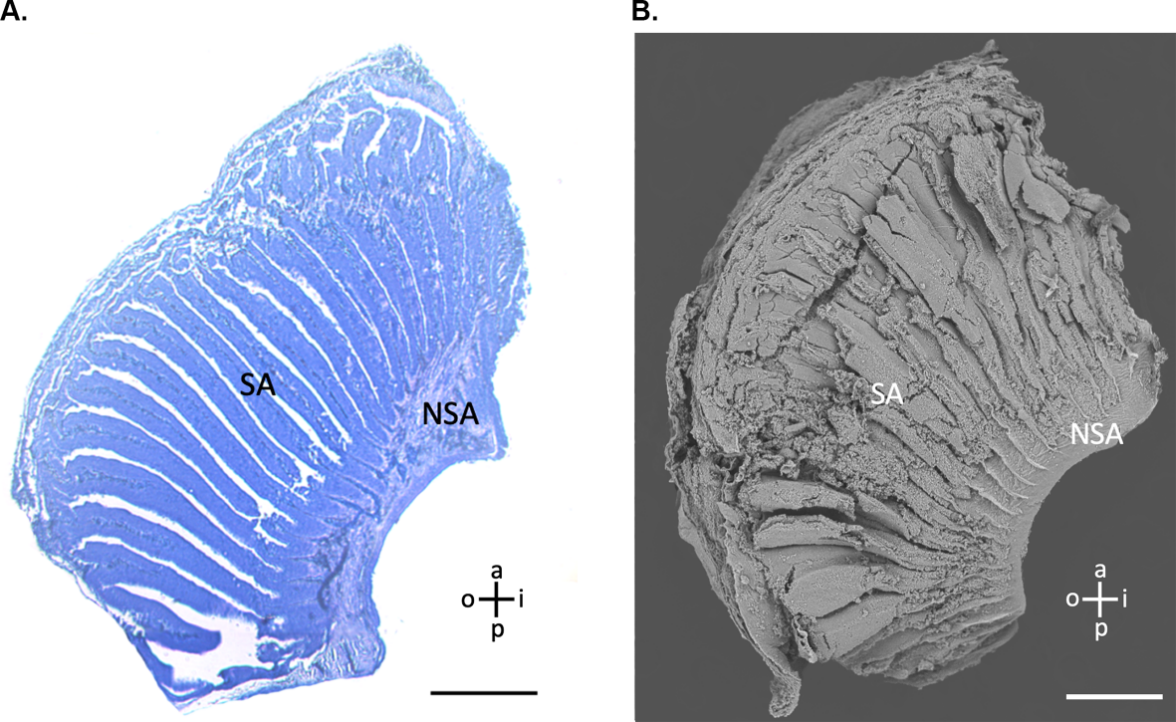
Definition of sensory area (SA; defined by ciliated supporting cells indicative of the sensory epithelium) vs. nonsensory area (NSA; defined by the nonsensory epithelium and superficial presence of the olfactory nerve layer). (**A**) Light micrographs of lamellae stained with toluidine blue (**B**) scanning electron micrographs of lamellae. Scale bars are 500 μm. Guide for lamella directional terminology showing anterior (a), posterior (p), inner (i), and outer (o).

**Fig. 5:**
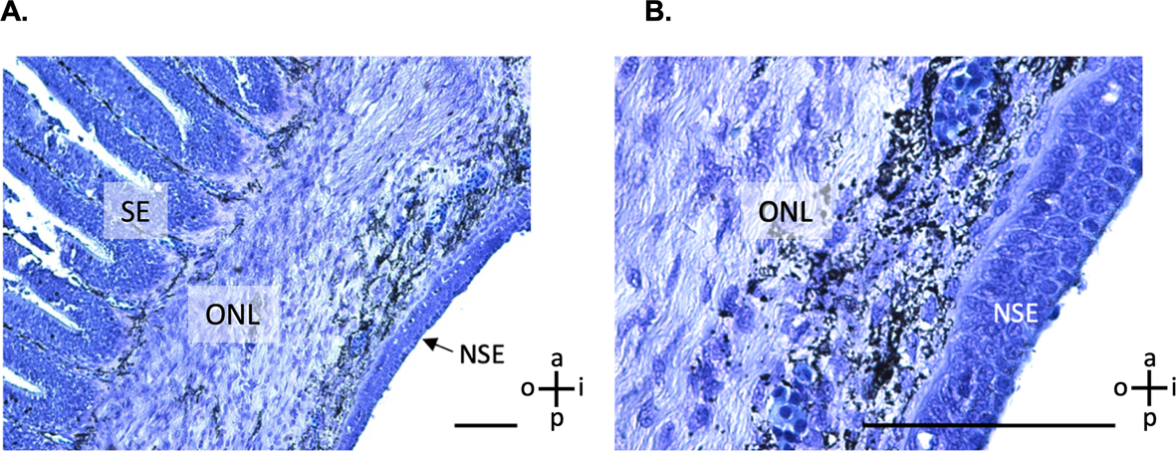
Light micrographs of the sensory epithelium, olfactory nerve layer, and nonsensory epithelium at low (**A**) and high magnification (**B**). SE: sensory epithelium, NSE: nonsensory epithelium, ONL: olfactory nerve layer. Scale bars 100 μm. Stained with toluidine blue. Guide for lamella directional terminology showing anterior (a), posterior (p), inner (i), and outer (o).

**Fig. 6:**
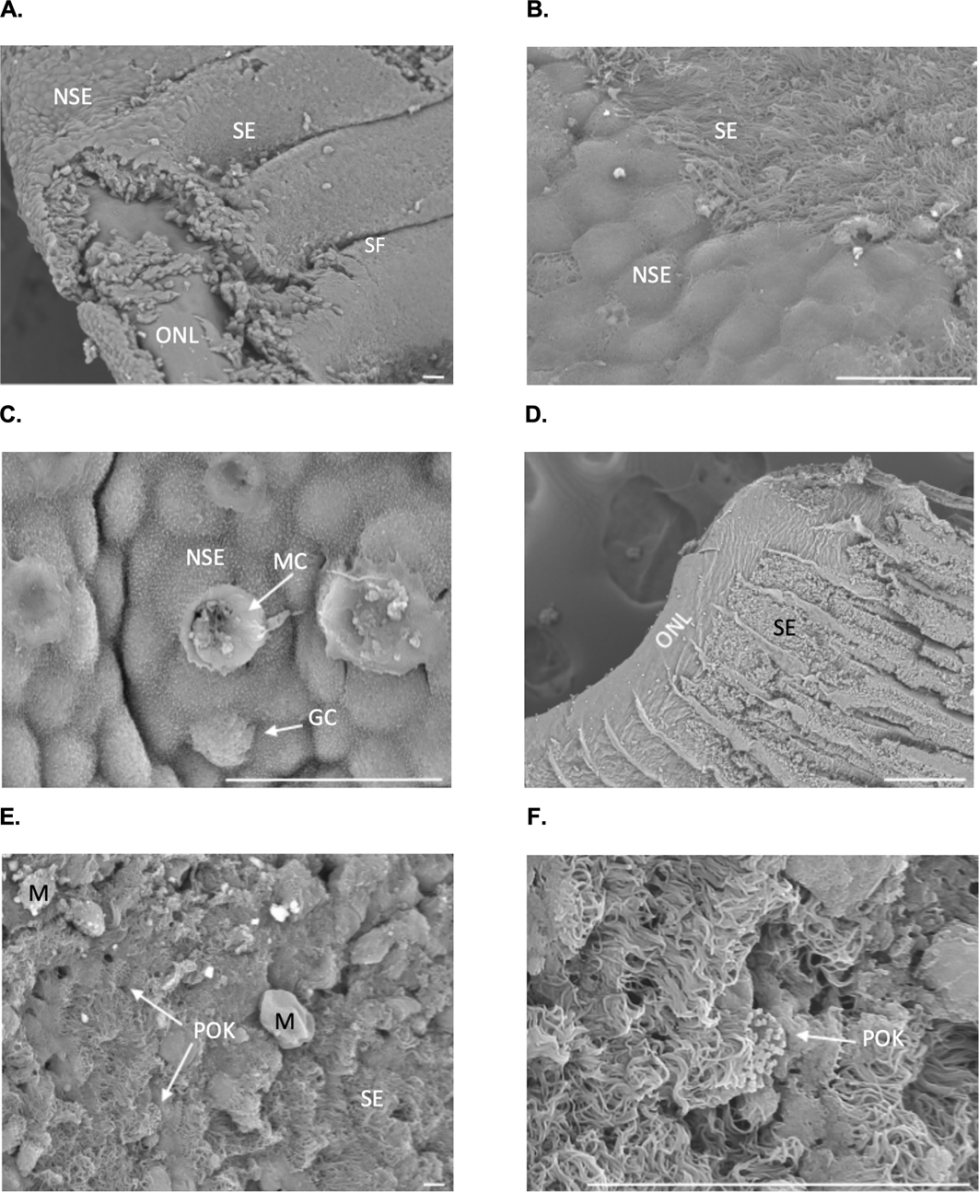
Scanning electron micrographs of the olfactory lamellae. (**A**) The nonsensory vs. sensory epithelium with secondary folding. Part of the nonsensory epithelium is removed, showing the olfactory nerve layer underneath. (**B**) A closer look at the nonsensory epithelium with microvilli and the sensory epithelium with cilia. (**C**) Nonsensory epithelium covered in microvilli with mucus cells and goblet cells. (**D**) The nonsensory epithelium has been removed, leaving the olfactory nerve layer. (**E**) Sensory epithelium with putative olfactory knobs. (**F**) Close up of a putative olfactory knob. GC: goblet cell, NSE: nonsensory epithelium, M: mucus, MC: mucus cell, ONL: olfactory nerve layer, POK: putative olfactory knob, SE: sensory epithelium, SF: secondary folds. Scale bars = 20 μm.

There was a maximum of 60 lamellae per olfactory rosette, numbered from 1 (the most lateral) to 60 (the most medial). For both SEM and LM images, three lamellar morphological metrics were collected along the lateral-to-medial gradient via ImageJ 1.52q (Schneider et al. 2012). First, we calculated the degree of secondary folding by counting the number of secondary folds for each lamella. Second, we calculated the percent sensory area for each lamella. The percent sensory area was the percentage of the total lamellar surface area covered by sensory epithelium (defined by densely packed ciliated supporting cells; Figs. 4, 5, and 6A and B). Alternatively, the nonsensory area was defined by the presence of either nonsenory epithelium or the olfactory nerve layer. For LM images, the most superficial sections were used, which showed both the nonsensory epithelium and the most superficial position of the olfactory nerve layer (Figs. 4 and 5). Finally, we calculated the relative surface area of each lamella. During SEM processing, the lamellae were dehydrated and shrank considerably. To account for this, as well as to compare between SEM and LM prepared lamellae, we measured the surface area of each lamella as a percentage of the lamella with the largest surface area to give us the relative surface area. Surface area measurements did not account for secondary folding. Each metric was calculated three separate times and the average of these three calculations was used. For statistical testing, both SEM and LM imaged lamellae were pooled for every 5 positions to get 12 groupings across the 60 positions. An ANOVA with Tukey post-hoc testing was performed on these 12 groupings to look for inter-positional distances. Because of small sample size, percent sensory area was not pooled or tested statistically.

Any lamellae without clear distinction of nonsensory vs. sensory epithelium were only measured for degree of secondary folding and relative surface area. Any lamellae with clearly defined secondary folds but damage to the outer edges were only used for degree of secondary folding. Both of these scenarios were particularly common for lamellae in the most lateral and the most medial positions within the rosette as they are smaller, more tightly packed, and seemed to be the most fragile. Additionally, the lamellae at these positions curved inwards, which did not allow us to obtain a useful cross-section of these lamellae histologically. These lamellae could only be sampled via extraction for SEM. In total, 11 LM lamellae images were used for all three metrics (degree of secondary folding, percent sensory area, and relative surface area). For SEM images, 78 were used for degree of secondary folding, 31 for percent sensory area, and 72 for relative surface area.

## Results

### Microstructure of the olfactory rosette

**Fig. 7:**
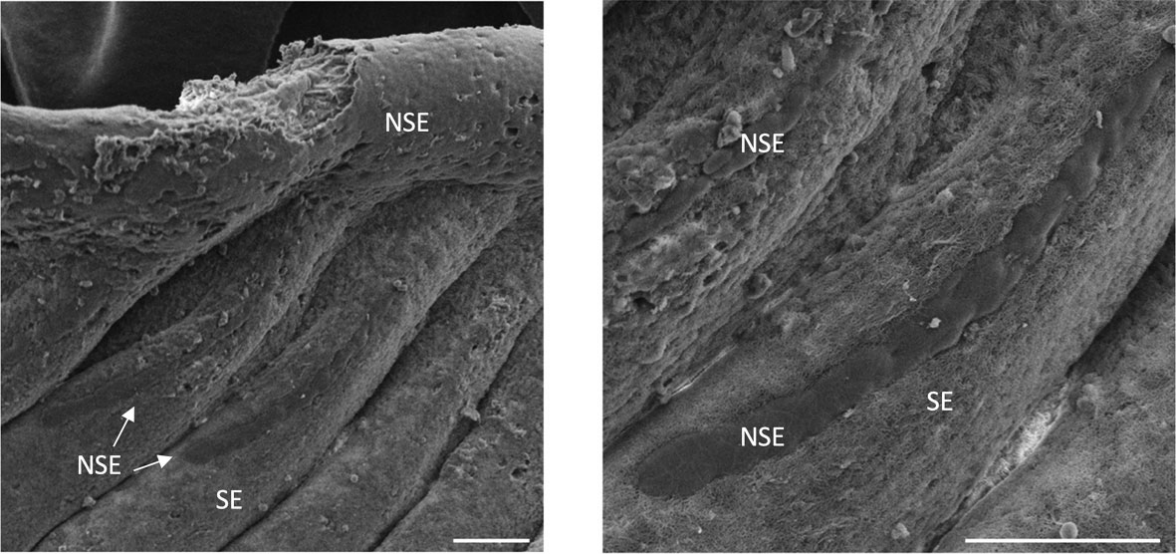
Projection of nonsenory epithelium into sensory epithelium. Only observed on one lamella and only on the last three folds on the posterior end of the lamella. Scale bars = 500 μm.

The bonnethead olfactory rosette is comprised of paired lamellae joined by a central raphe which attaches to the olfactory bulb (Fig. 2A). Overall, the cross-section appears as a “horse-shoe” shape. The space between the paired lamellae forms the incurrent chamber, and a gap at the posterior end of the lamellae forms the excurrent chamber. The nonsensory epithelium is concentrated around the inner margins of the lamellae (Figs. 5 and 6A). Right underneath the nonsensory epithelium is the olfactory nerve layer (Figs. 5 and 6A and D). The nonsensory epithelium is covered in microvilli and contains both goblet and mucus cells (Fig. 6B–C). The sensory epithelium is covered in ciliated support cells and has secondary folds that increase the surface area (Fig. 5B). We did not observe typical olfactory knobs, the dendritic swellings of olfactory receptor cells, reported in other shark species (Schluessel et al. 2008; Theiss et al. 2009; Camilieri-Asch et al. 2020a). However, we did observe putative olfactory knobs *sensu*Schluessel et al. (2008) in areas of sensory epithelium with lower cilia density (Fig. 6E and F). While these are similar to previously published SEMs of olfactory knobs, because of the visual differences and lack of histological support, we have labeled them as putative olfactory knobs. Oddly, on one lamella in one rosette in one shark, we observed fingerlike projections of the nonsensory epithelium into the sensory epithelium (Fig. 7).

**Fig. 8:**
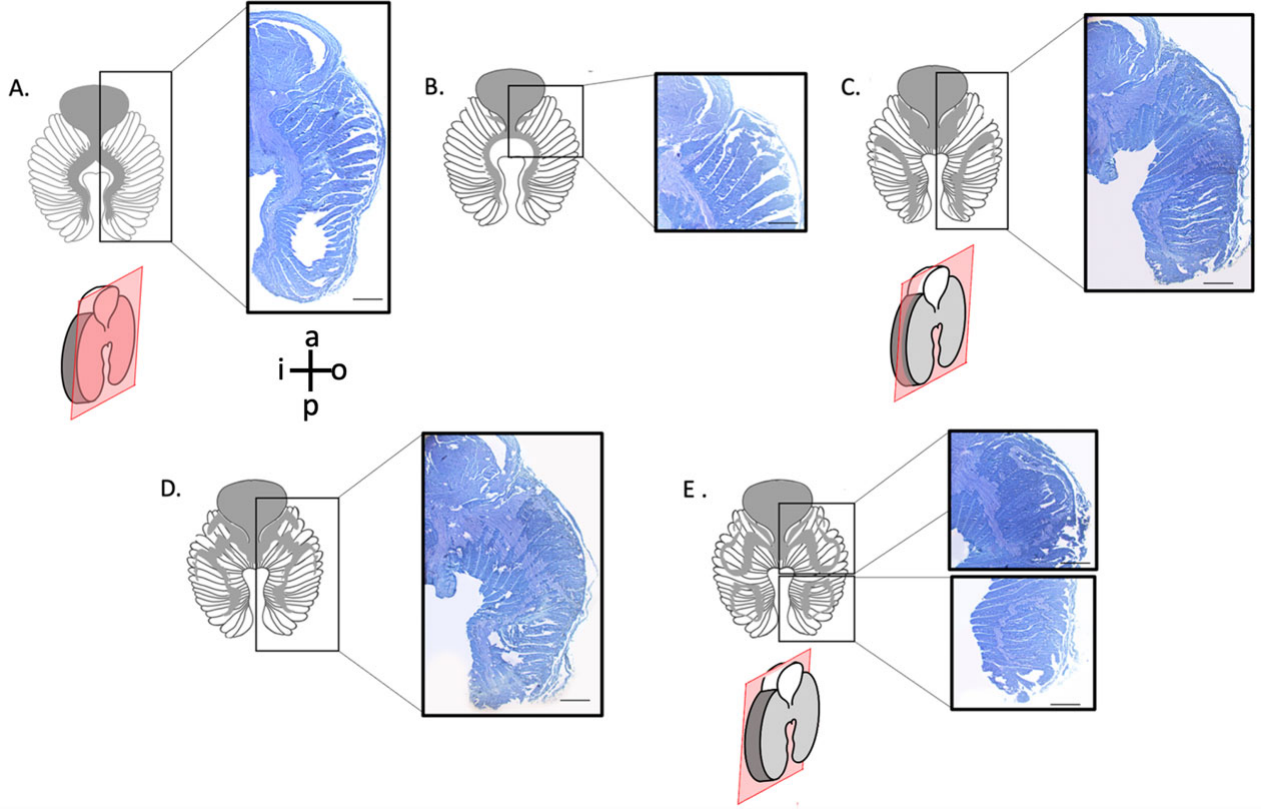
Lamellar microstructure. Serial sections through a single centrally positioned lamella (**A-E**) showing the olfactory nerve layer (stained lighter blue in histological sections) innervating the lamella. Grey shading indicates the olfactory nerve layer projections in each section. Pink box indicates where in the lamella the section came from. Scale bars = 500 μm. Stained with toluidine blue. Guide for lamella directional terminology showing anterior (a), posterior (p), inner (i), and outer (o).

We visually mapped the path of the olfactory nerve layer through serial sections through the lamellae (Fig. 8). In the first of the serial sections, the olfactory nerve runs from the bulb, along the inner margins of each lamella and branches out into the middle of the secondary folds (Fig. 8A). This can also be observed in Fig. 5 where the black staining of the myelin sheath of the large diameter nerves gives rise to narrow nerves running into each lamella. Later in the serial sections, the nerves travel anteriorly toward the bulb (Fig. 8B). As we continue sectioning through the lamella, the anterior part of the olfactory nerve layer covers more area while the posterior part expands toward the outer margins, beginning to form a semicircle (Fig. 8C). Next, the anterior and posterior nerves rejoin halfway between the inner and outer margins. Finally, at the last serial section, the olfactory nerve layer forms two loops at the anterior and posterior ends of the lamellae (Fig. 8D). This pattern was observed in all sectioned lamellae, regardless of their position within the rosette.

### Morphological differences along the medial-to-lateral gradient

**Fig. 9:**
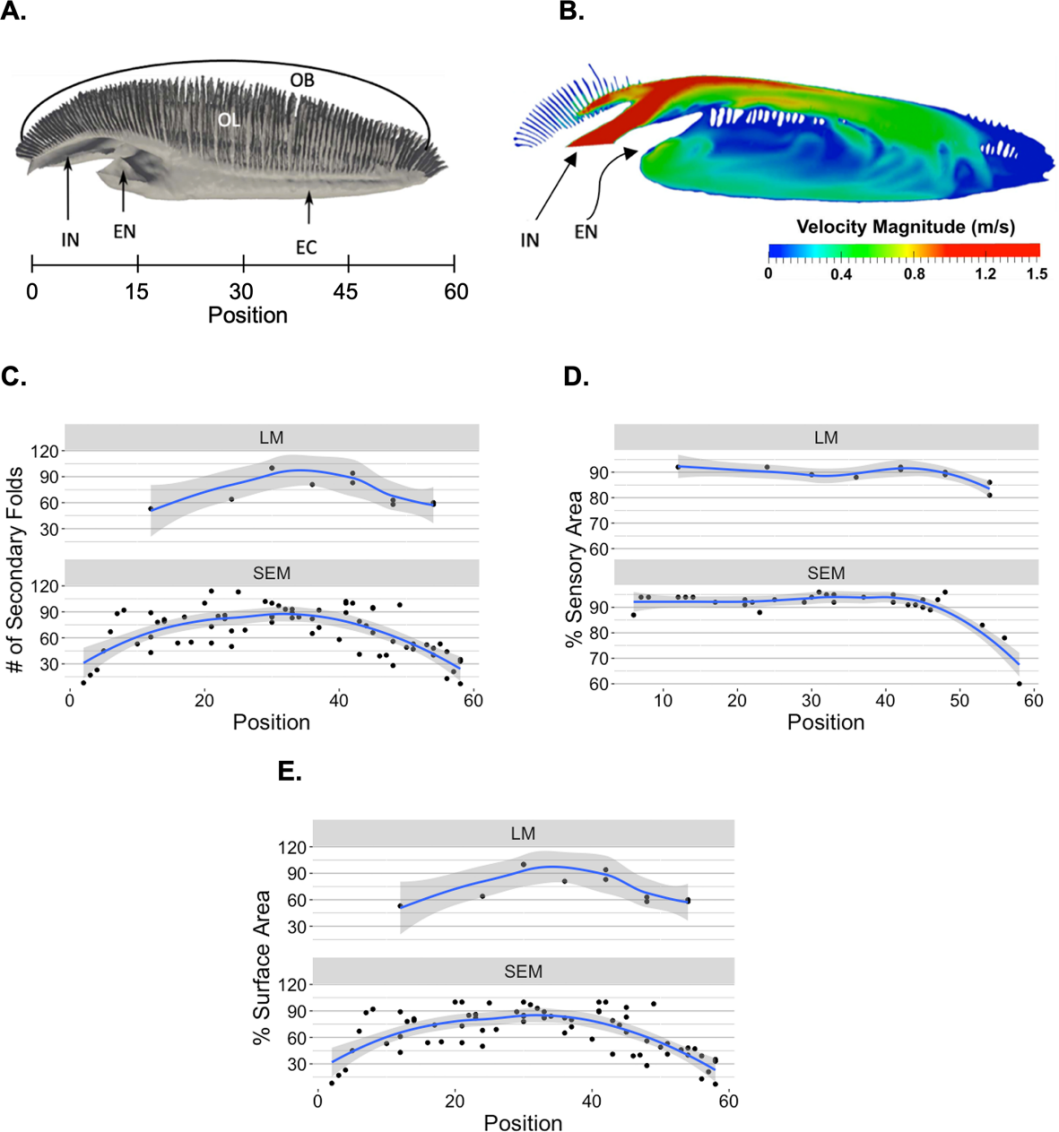
Lamellar morphological patterns throughout the rosette. (**A**) 3D anatomical model and (**B**) calculated velocity fields (fine mesh) of smalleye hammerhead olfactory rosettes from Rygg et al. 2013. Lamellar position is defined along the lateral-to-medial gradient from 1 (most lateral) to 60 (most medial). Trends in calculated morphological metrics for lamellae imaged through both LM and SEM, such as (**C**) degree of secondary folding, (**D**) percent sensory area, and (**E)** relative surface area standardized as a percentage of the largest lamella, are visualized with a LOESS (locally weighted smoothing) smooth curve and a shaded 95% confidence interval. Each dot represents one lamella. All measured surface areas did not account for secondary folding. EC: excurrent channel, EN: excurrent naris, IN: incurrent naris, OB: olfactory bulb, OL: olfactory lamellae.

**Fig. 10:**
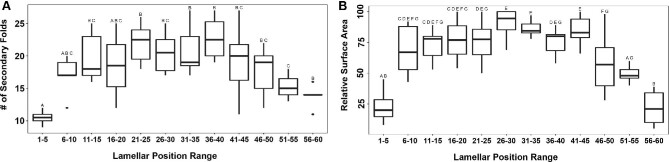
Boxplots showing significant differences in (**A**) degree of secondary folding and (**B**) relative surface area for lamellae binned by position. Letters above each box represent the results of pair-wise comparisons using Tukey post-hoc testing. For each binned position, boxes not sharing a letter are significantly different (*P* < 0.05). Because of low sample size, percent sensory area data was not binned or tested statistically. All measured surface areas did not account for secondary folding.

We observed morphological variation in lamellae based on their positioning within the rosette (Fig. 9). These findings were consistent for both LM and SEM imaged lamellae. Degree of secondary folding decreased at either extreme with fewer folds counted on the lamellae from the lateral (lamellar positions closer to 1) and medial (positions closer to 60) ends of the rosette and more folds counted within the central lamellae (positions closer to 30; Fig. 9C). Looking at binned data, lamellae at lateral positions 1–5 and medial positions 51–60 had significantly less (*P* < 0.05) secondary folds than central lamellae at positions 31–40 (Fig. 10A). Due to the lower sample size, we were unable to perform statistical tests on percent sensory area data. However, we still observed a strong trend: the percentage of the sensory area remained relatively consistent throughout the majority of the rosette, ranging between 96–87% of the total area. However, the medial lamellae show a decrease in percentage, reaching a low of 60% for the most medial lamella (Fig. 9D). Finally, lamellae within the center of the rosette had higher relative surface areas than lamellae at the lateral or medial ends (Fig. 9E). For binned data, lamellae at lateral positions 1–5 and medial positions 56–60 had significantly lower relative surface areas (*P* < 0.05) than all other lamellae (Fig. 10B).

## Discussion

Bonnethead sharks exhibit similar lamellar morphology to other sharks. Their olfactory lamellae (OL) are covered with nonsensory epithelium on the inner margins and sensory epithelium extending to the outer margins (Figs. 4–6). We found sensory epithelium in both the “troughs” and “peaks” of the secondary folds, similar to spiny dogfish (*Squalus acanthias*) and small-spotted catsharks (*Scyliorhinus canicular*; Theisen et al. 1986). The only other published SEM of a sphrynid shark (scalloped hammerheads, *Sphyrna lewini*) reported projections of the nonsensory epithelium into the sensory epithelium, which was only observed in one lamella in one rosette from one shark in this study (Fig. 7; Schluessel et al. 2008). These lamellae were located in the center region of the rosette (position 45) and only had these projections on the last 3 secondary folds on the posterior end.

Secondary folds within the lamellae are covered in dense ciliated supporting cells. Unlike teleosts, elasmobranchs do not possess ciliated receptor cells. Instead, they have microvillus and crypt ORNs (Northcutt 1978; Theisen et al. 1986; Zeiske et al. 1987; Takami et al. 1994; Hansen and Zielinski 2005; Ferrando et al. 2006; Schluessel et al. 2008; Theiss et al. 2009; Camilieri-Asch et al. 2020a). Some sharks possess olfactory knobs, which are the dendrites of microvillus ORNs that reach the epithelial surface (Theisen et al. 1986; Zeiske et al. 1987; Schluessel et al. 2008; Theiss et al. 2009; Camilieri-Asch et al. 2020a). We did not observe these exact structures in bonnethead lamellae. The supporting ciliated cells were dense, which may have obscured these structures from view. Previous SEMs of scalloped hammerheads also did not report olfactory knobs (Schluessel et al. 2008). However, we did find structures within areas of sparser cilia coverage, which we labeled putative olfactory knobs (Fig. 5E and F). These structures were similar morphologically but not enough for us to definitively label them olfactory knobs. It is possible that this could be a variant morphology of olfactory knobs, but the function of these structures remains unknown.

To our knowledge, this is the first time the olfactory nerve layer, which contains the axons projecting from the ORNs to the bulb, has been visually tracked through a shark lamella. Previous work has focused on the projection of the ORNs in the bulb, not through the lamellae. The relationship between the ORN axons and the olfactory bulb has been described for only a handful of elasmobranch species. In most vertebrates, ORN axons project to differential locations within the olfactory bulb according to the odorant class for which they bind (Friedrich and Korsching 1998; Xu et al. 2000; Nikonov and Caprio 2001; Hamdani and Døving 2007). Some elasmobranchs exhibit a topographic arrangement within their olfactory bulb, similar to teleosts (Nikonov and Caprio 2001; Sato and Suzuki 2001; Hansen et al. 2003, 2004; Hansen and Zielinski 2005; Døving et al. 2011). In spotted catsharks (*S. canicula*), for example, crypt ORNs correspond to ventral glomeruli while microvillus ORNs project in numerous axon bundles within the remainder of the glomeruli (Ferrando et al. 2009). However, other elasmobranchs, such as bonnetheads, have somatotopically arranged olfactory bulbs, with each ORN projecting to the olfactory bulb glomeruli immediately anterior to it (Dryer and Graziadei 1993; Meredith et al. 2013). Whether this somatotopic arrangement continues through the peduncle and into the telencephalon remains to be investigated. Similarly, whether the location of the ORN or the type of ORN impact where its axons travel within the olfactory nerve layer is still unknown. We did not observe single ORN axons, just the general pathway of the nerve fibers through the lamella. In other words, we can describe the highway of information from the lamellar ORNs to the olfactory bulb, but not the individual paths along that highway. While we are not able to determine from our data whether the olfactory never layer in the lamellae is topographically or somatotopically arranged, describing then general pathway is the first step in exploring these possible connections.

We report differences in degree of folding, percent sensory area, and relative surface area of lamellae along the lateral-to-medial gradient within the rosette of bonnethead sharks. Medial lamellae have fewer secondary folds and less sensory surface area than centrally or laterally located lamellae (Figs. 9C and D and 10A). Rygg et al. (2013) described the medial portion of the hammerhead olfactory organ as a near-stagnant, recirculating area. The results suggest that the fact that lamellae in this region that have less surface area dedicated to olfactory sensation is possibly correlated with slower velocity and stagnation at this part of the organ. Although previous physiological work has not found a correlation between higher olfactory sensitivity and larger lamellar surface area, we suggest that amount of sensory epithelium may predict sensitivity similar to how axon morphology or density is also used as a proxy for sensitivity (George and Holliday 2013; Jones and Marshall 2019). Additionally, we only found secondary folds within the sensory epithelium, so the degree of secondary folding directly correlates with sensory surface area. Furthermore, physiological testing using electro-olfactorgrams record a summation of action potentials in the area that the electrode is placed. Our findings suggest that where the electrode is placed along the rosette may impact readings of olfactory sensitivity.

While Rygg et al.’s (2013) model was based on the smalleye hammerhead (*S. tudes*) olfactory system, bonnethead sharks possess a similar olfactory morphology. We suggest that a computational fluid dynamics model be produced to confirm that these flow patterns are conserved within the bonnethead olfactory organ. A relationship between slower water velocities and possible lower sensor density makes sense-slower, recirculating flows allows chemicals in the water to have more time to bind to ORNs. Conversely, at high velocities, chemicals are passing over the sensory epithelium quickly and may have a lower chance to bind to a sensor. This disadvantage of higher flow can be combatted by more sensory surface area with higher densities of ORNs. Although higher degrees of secondary folding and less sensory area may indicate more area for ORNs and therefore more sensitivity, physiological testing of olfactory sensitivity along this lateral-to-medial gradient need to be conducted to confirm our hypotheses.

Additionally, we observed differences in lamellar relative surface area with the larger lamellae occurring in the center of the olfactory organ (Figs. 9E and 10B). These lamellae may be more robust to withstand the higher flow velocities experienced in most of the rosette. Lamellae in the medial region were smaller but likely experience lower velocities. Lamellae were also smaller in the lateral portion of the organ than in the center; however, the flow around these lamellae has not been modeled in any species. In Rygg et al. (2013), these lateral most lamellae (positions ∼1–10) were not included in the computational fluid dynamics models. The flow within the rosette at this location should also be modeled to inform the relationship between lamellar morphometrics and water flow.

Despite differences in the number of secondary folds based on positioning, we observed secondary folds through the entirety of the lamellae regardless of positioning within the rosette. This pattern is shared by most elasmobranchs. However, lemon sharks (*Negapiron brevirostris*) and clearnose skates (*Raja elganteria)* lack secondary folding on the posterior end of centrally located lamellae (Takami et al. 1994; Meredith et al. 2013). Additionally, loss of secondary folding in the posterior end of the lamellae has been reported for brown-banded bamboo sharks (*Chiloscyllium punctatum*) but the specific position of these lamellae was not reported (Schluessel et al. 2008). Additionally, whether the sensory epithelium continues posteriorly despite the loss of secondary folds was not explicitly stated for lemon or bamboo sharks. The functional reason for these variations remains unknown.

Previous work has tied differences in olfactory morphology among elasmobranch species to ecology rather than evolutionary relationships. Yopak et al. (2015) found that sharks living in pelagic-coastal and oceanic environments had the largest olfactory bulbs while reef-associated sharks had the smallest. Similarly, Schluessel et al. (2008) reported that bentho-pelagic elasmobranchs had more lamellae, larger percentages of sensory area, and larger rosettes when correlated to body length than benthic species. They also reported a diet effect; elasmobranchs that consumed echinoderms and mollusks had higher bulb mass than those that consumed crustaceans. However, for the lamellar measurements, they did not specify where the analyzed lamellae were positioned along the lateral-to-medial gradient, which our results suggest may impact both the size of the lamellae and the amount of sensory area.

In summary, we found that olfactory lamellae in areas of the olfactory organ that experience faster water flow have a larger percentage of sensory area and more secondary folds compared to those exposed to lower water velocities. Our work implies that sensory morphology and possibly sensitivity within the organ change in correlation with water flow. Differences along the lateral-to-medial gradient within the olfactory rosette should be taken into account in future studies of olfactory morphology, especially for sharks with elongated olfactory organs, such as sphrynids. Sharks with differently shaped olfactory organs may produce different flow patterns and exhibit different configurations of lamellar morphologies. It is important to not only generate computational fluid dynamics to understand fluid flow for differently organized shark olfactory organs, but also investigate potential differences in sensitivities within the organ. A combination of morphology, physiology, and fluid dynamics, as well as additional comparative data in a wider variety of species, will lead to a better understanding of how shark olfactory organs sample chemical stimuli in their environment and the diversity of neuroecology of sharks and their sensory systems.

## Data Availability

Data generated by this study and supporting the conclusions of this article will be made available by authors upon request without undue reservation.
